# Molecular Biology of Histamine System, Volume 1

**DOI:** 10.3390/ijms23095026

**Published:** 2022-04-30

**Authors:** Paul Chazot

**Affiliations:** Department of Biosciences, University of Durham, Durham DH1 3LE, UK; paul.chazot@durham.ac.uk

Histamine is arguably the most pleiotropic transmitted in the human body. It is released by both professional (mast cells, neurons) and non-professional cells (bone marrow leukocytes), and it imparts its many physiological actions through binding four G-protein-binding receptors (GPCRs, H1-4Rs). Despite being recognised for over a century, there is still more to be discovered about the molecular, structural, and functional properties of the histamine system and its potential for therapeutic exploitation. This volume has provided interesting new detailed insights into the histamine system, using a range of classic and state-of-the art methodologies.

The Exome Aggregation Consortium dataset for GPCR proteins has revealed a total of 463 naturally occurring genetic missense variations in the histamine receptor family. This offers a plethora of opportunities to the field to probe the key molecular structural elements of the histamine receptor family. Four missense variants R1273.52 × 52H, R13934.57 × 57H, R4096.29 × 29H, and E4106.30 × 30K, were selected for the histamine H1 receptor (H1R) to be explored in detail as a proof-of-concept. The data accrued from the study supported the hypothesis that the E4106.30 × 30K mutation shifts the equilibrium of the H1R binding towards active conformations. The study of these selected missense variants gives additional new insight into the structural basis of H1R activation and, moreover, highlights that missense variants can result in pharmacologically distinct consequences as compared to wild-type receptors and should consequently be considered in the drug discovery process for the whole histamine receptor family [1]. 

In terms of ligand-binding determinants and kinetics, the relationship between the kinetic and thermodynamic binding properties of antihistamines was examined in another study, followed by an evaluation of the structural determinants responsible for their kinetic binding properties using quantitative structure–activity relationship (QSAR) analyses. A non-invasive dynamic mass redistribution (DMR) assay designed for the human H1–4Rs expressed in HEK cells was reported, showing excellent signal-to-background ratios for both endogenous histamine and for synthetic inverse agonists [2].

Kinetic analyses of the rate constants of association and dissociation (kon and koff, respectively) of antihistamines have suggested that second-generation antihistamines have a long duration of action owing to the long residence time (1/koff) at the H1 receptors. This is a hot area of pharmacological research, pioneered by histamine receptorologists. Kinetic binding properties using QSAR analyses showed that although the binding enthalpy and entropy might contribute to the increase and decrease, respectively, in the koff values, there was no significant relationship with the kon values [3].

Bilastine is a non-sedating, centrally sparing, and long-acting histamine antagonist therapeutic with selective peripheral H1 receptor antagonist affinity and little or no affinity for muscarinic receptors, much studied over recent years. A study herein evaluated the roles of two key residues Lys179ECL2 and Lys1915.39 in regulating the electrostatic and hydrophobic binding of bilastine to H1 receptors by thermodynamic analyses. The binding enthalpy and entropy of bilastine were estimated from the classic van’t Hoff equation using dissociation constants [4].

Using a novel combination of cellular experimental assays and Gaussian accelerated molecular dynamics (GaMD) simulations, the coupling profiles of the H2R and H4R to engineered mini-G proteins (mG) was explored. Due to the specific residue interactions, all mG α5 helices of the H2R complexes adopted the Gs-like orientation toward the receptor transmembrane (TM) 6 domain, whereas, in H4R complexes, only mGi was in the Gi-like orientation toward TM2, which was in agreement with the respective Gs- and Gi-coupled GPCRs structures resolved by X-ray/cryo-EM [5]. Further studies with the H1R, and in particular the H3R, are needed and, importantly, confirmation of the respective coupling partners in native tissues.

In terms of histamine cell signalling, new insights were gathered regarding skin molecular biology. The chemokine CCL18 is produced in cells of the myelomonocytic lineage (Th2 cells attracting chemokine) and represents one of the most highly expressed chemokines in the lesional skin and serum of atopic dermatitis patients. A new function of histamine was reported showing upregulation of the CCL18 in activated human M2 macrophages. This is proposed as a candidate to influence the course of atopic dermatitis and, therefore, the development of new respective therapeutic interventions [6]. I would concur that this would be worth pursuing and is likely H4R-mediated. Furthermore, a detailed new review discussed the involvement of histamine and H4R in inflammatory and inflammation-associated diseases of the gut. Ligands at H4R are still being tested pre-clinically and in the clinical trials of a range of inflammatory diseases. These trials, however, documented only modest beneficial effects of H4R ligands so far, leading to many pharma programmes being put on hold. Nevertheless, pre-clinically, H4R is still the subject of ongoing research, analysing various inflammatory, allergic, and autoimmune diseases. During inflammatory reactions in gut tissues, including diabetes, histamine concentrations rise in affected areas, indicating its possible biological effect. Indeed, in histamine-deficient mice, experimentally induced inflammation of the gut is reduced in comparison to that in histamine-competent mice. These studies encourage the continued development of H4R-targeted therapies in this domain [7]. In another important therapeutic domain, the expression of H4R isoforms in three human aggressive T-cell lymphomas was reported. With regards to further therapeutic potential, but from a different pharmacological perspective, histamine and specific H4R agonists (VUF8430 and JNJ28610244) significantly reduced cell viability in these cells, evidencing the chemotherapeutic potential of H4R agonists [8]. 

Overall, this themed edition took us on a journey from advances in understanding ligand binding at the receptor level, through G-protein coupling partners, new cell signalling pathways, and, thence, new therapeutic indications for the histamine receptor family, particularly the H4R family. The author declares that volume 2 will follow soon.

## References

[1] Ma X., Segura M.A., Zarzycka B., Vischer H.F., Leurs R. (2021). Analysis of Missense Variants in the Human Histamine Receptor Family Reveals Increased Constitutive Activity of E410^6.30 × 30^K Variant in the Histamine H_1_ Receptor. Int. J. Mol. Sci..

[2] Seibel-Ehlert U., Plank N., Inoue A., Bernhardt G., Strasser A. (2021). Label-Free Investigations on the G Protein Dependent Signaling Pathways of Histamine Receptors. Int. J. Mol. Sci..

[3] Akimoto H., Uesawa Y., Hishinuma S. (2021). Molecular Determinants of the Kinetic Binding Properties of Antihistamines at the Histamine H_1_ Receptors. Int. J. Mol. Sci..

[4] Akimoto H., Sugihara M., Hishinuma S. (2021). Differential Regulation of Bilastine Affinity for Human Histamine H_1_ Receptors by Lys 179 and Lys 191 via Its Binding Enthalpy and Entropy. Int. J. Mol. Sci..

[5] Höring C., Conrad M., Söldner C.A., Wang J., Sticht H., Strasser A., Miao Y. (2021). Specific Engineered G Protein Coupling to Histamine Receptors Revealed from Cellular Assay Experiments and Accelerated Molecular Dynamics Simulations. Int. J. Mol. Sci..

[6] Mommert S., Schaper J.T., Schaper-Gerhardt K., Gutzmer R., Werfel T. (2021). Histamine Increases Th2 Cytokine-Induced CCL18 Expression in Human M2 Macrophages. Int. J. Mol. Sci..

[7] Schirmer B., Neumann D. (2021). The Function of the Histamine H4 Receptor in Inflammatory and Inflammation-Associated Diseases of the Gut. Int. J. Mol. Sci..

[8] Clauzure M., Táquez Delgado M.A., Phillip J.M., Revuelta M.V., Cerchietti L., Medina V.A. (2022). Histamine H4 Receptor Agonism Induces Antitumor Effects in Human T-Cell Lymphoma. Int. J. Mol. Sci..

